# Predicting Fluctuations in Cryptocurrency Transactions Based on User Comments and Replies

**DOI:** 10.1371/journal.pone.0161197

**Published:** 2016-08-17

**Authors:** Young Bin Kim, Jun Gi Kim, Wook Kim, Jae Ho Im, Tae Hyeong Kim, Shin Jin Kang, Chang Hun Kim

**Affiliations:** 1 Interdisciplinary Program in Visual Information Processing, Korea University, Seoul, Korea; 2 School of Games, Hongik University, Seoul, Korea; 3 Department of Computer and Radio Communications Engineering, Korea University, Seoul, Korea; East China University of Science and Technology, CHINA

## Abstract

This paper proposes a method to predict fluctuations in the prices of cryptocurrencies, which are increasingly used for online transactions worldwide. Little research has been conducted on predicting fluctuations in the price and number of transactions of a variety of cryptocurrencies. Moreover, the few methods proposed to predict fluctuation in currency prices are inefficient because they fail to take into account the differences in attributes between real currencies and cryptocurrencies. This paper analyzes user comments in online cryptocurrency communities to predict fluctuations in the prices of cryptocurrencies and the number of transactions. By focusing on three cryptocurrencies, each with a large market size and user base, this paper attempts to predict such fluctuations by using a simple and efficient method.

## Introduction

The ubiquity of Internet access has triggered the emergence of currencies distinct from those used in the prevalent monetary system. The advent of cryptocurrencies based on a unique method called “mining” has brought about significant changes in the online economic activities of users. Various cryptocurrencies have emerged since 2008, when Bitcoin was first introduced [1, 2]. Nowadays, cryptocurrencies are often used in online transactions, and their usage has increased every year since their introduction [3, 4].

Cryptocurrencies are primarily characterized by fluctuations in their price and number of transactions [2, 3]. For instance, the most famous cryptocurrency, Bitcoin, had witnessed no significant fluctuation in its price and number of transactions until the end of 2013 [3], when it began to garner worldwide attention, and witnessed a significant rise and fluctuation in its price and number of transactions. Other cryptocurrencies—Ripple and Litecoin, for instance—have shown significantly unstable fluctuations since the end of December 2013 [5]. Such unstable fluctuations have served as an opportunity for speculation for some users while hindering most others from using cryptocurrencies [2, 6, 7].

Research on the attributes of cryptocurrencies has made steady progress but has a long way to go. Most researchers analyze user sentiments related to cryptocurrencies on social media, e.g., Twitter, or quantified Web search queries on search engines, such as Google, as well as fluctuations in price and trade volume to determine any relation [8–12]. Past studies have been limited to Bitcoin because the large amount of data that it provides eliminates the need to build a model to predict fluctuations in the price and number of transactions of diverse cryptocurrencies.

Therefore, this paper proposes a method to predict fluctuations in the price and number of transactions of cryptocurrencies. The proposed method analyzes user comments on online cryptocurrency communities, and conducts an association analysis between these comments and fluctuations in the price and number of transactions of cryptocurrencies to extract significant factors and formulate a prediction model. The method is intended to predict fluctuations in cryptocurrencies based on the attributes of online communities.

Online communities serve as forums where people share opinions regarding topics of common interest [13–17]. Therefore, such communities mirror the responses of many users to certain cryptocurrencies on a daily basis. Cryptocurrencies are largely traded online, where many users rely on information on the Web to make decisions about selling or buying them [4, 18]. In this paper, daily topics and relevant comments/replies in cryptocurrency communities are analyzed to determine how the opinions of community users are associated with fluctuations in the price and number of transactions of cryptocurrencies on a daily basis.

The proposed method is applicable to a range of cryptocurrencies, and can predict fluctuations in the prices of such cryptocurrencies as Bitcoin, Ripple, and Ethereum to a certain extent (approximately 74% weighted average precision). Moreover, the rise and fall in the number of transactions of Bitcoin and Ethereum can be predicted to some extent.

## Methods

### System Overview

**Fig 1:**
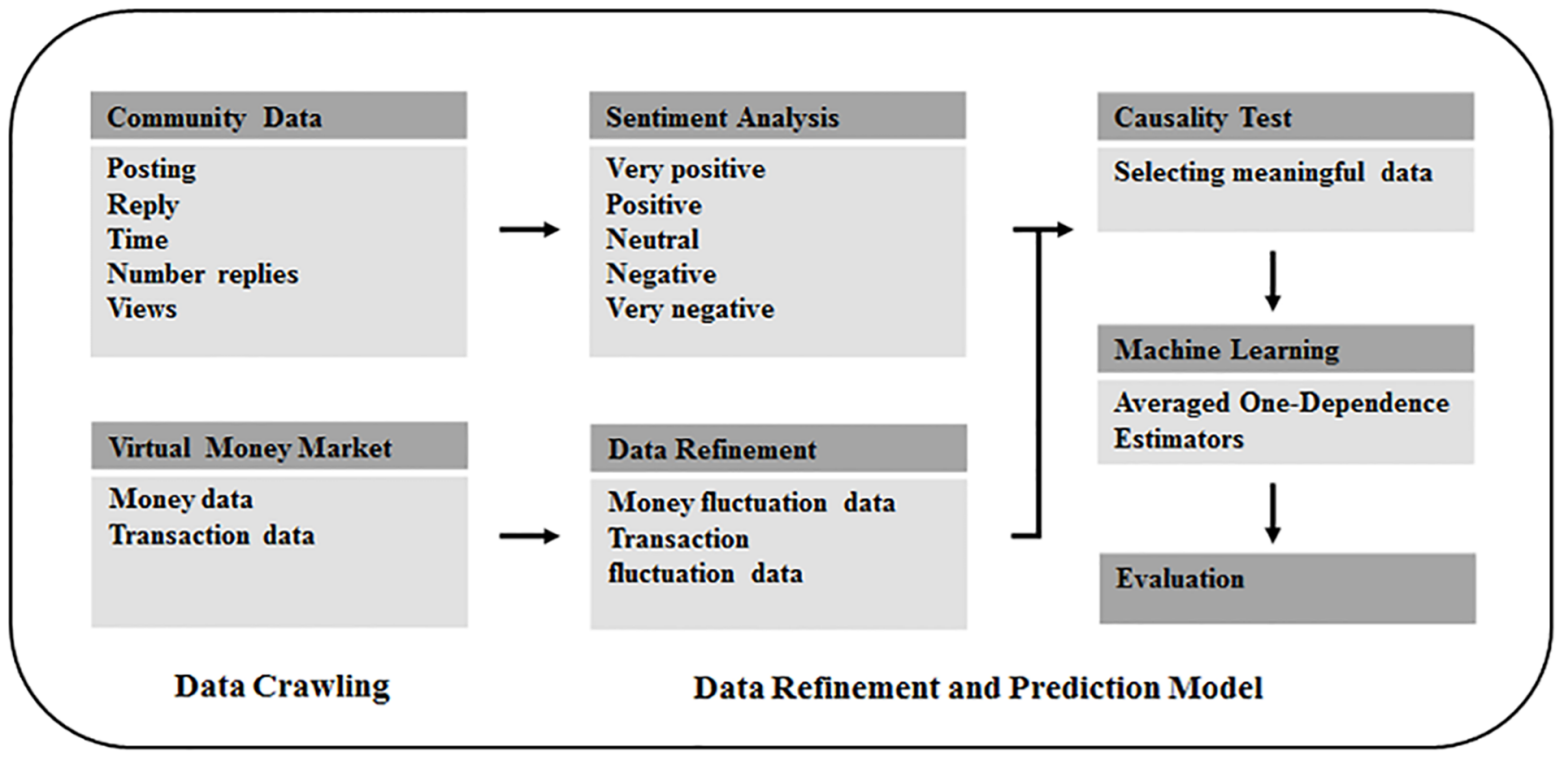
System overview.

For the proposed system, we crawled all comments and replies posted in online communities relevant to cryptocurrencies [19–21]. We then analyzed the data (comments and replies) and tagged the extent of positivity or negativity of each topic as well as that of each comment and reply. Following this, we tested the relation between the price and number of transactions of cryptocurrencies based on user comments and replies to select data (comments and replies) that showed significant relation. Finally, we created a prediction model via machine learning based on the selected data to predict fluctuations (Fig 1).

### Crawling user comment data

We crawled data needed to create the prediction model. Once the environment for cryptocurrency trading among users is established, transactions between users lead to fluctuations in price [4]. We hypothesized that user comments in certain online cryptocurrency communities may affect fluctuations in their price and trading volume. Thus, we crawled the relevant data. Approximately 670 types of cryptocurrencies existed as of February 2016 [22]. Of the available ones, we crawled online communities for the top three in terms of market cap, i.e., Bitcoin, Ethereum, and Ripple. We did not include Litecoin in this study because its online communities seemed not to be sufficiently active to be considered in this experiment, despite its large market cap and broad user base.

Since Bitcoin was the first cryptocurrency, it has a large user community. In the Bitcoin community [19], data items were collected starting from December 2013, when the cryptocurrency became widely available. In the Ethereum community [20], data were collected from August 7, 2015, since when the community stabilized to the extent that at least one topic has since been posted every day and transaction data are available. From the Ripple community [21], all data since the creation of the community were gathered. In all communities of interest, we collected data in a legitimate manner, in compliance with their terms and conditions. Moroever, the collected data did not involve any personally identifiable information.

The cryptocurrencies of interest in this paper had online communities where users shared opinions on the relevant topics. The Bitcoin community [19] is divided into four sections, i.e., a “Bitcoin” section on Bitcoin-related topics, an “Economy” section on transactions, an “Alternate cryptocurrencies” section concerning other cryptocurrencies, and an “Other” section for other topics. Each section has three-five subsections. The “Bitcoin” section consisted of “Bitcoin Discussion,” “Development & Technical Discussion,” “Mining,” “Technical Support,” and “Project Development.” The “Alternate cryptocurrencies” section had a similar structure. For this paper, we crawled the discussion sub-sections for topics related to each of the cryptocurrencies.

Comments and relevant replies posted by users on bulletin boards in each community were crawled. Furthermore, the time when each comment and replies to it were posted, the number of replies to each comment, and the number of views were crawled as well. Replies quoting previous comments and replies were crawled excluding overlapping sentences. Each community’s HTML page was crawled using Python [23]. Using Python’s regex, we parsed the tags on HTML pages to extract the number of topics, the number of replies, the dates on which the topics and replies were posted, and the URL of each topic from the bulletin boards. Based on the URLs of extracted topics, their contents and replies to them were extracted. The extracted topics, the dates on which they were posted, topic contents, reply contents, and reply dates were saved in .json format, which was in turn converted into other formats (e.g. csv) appropriate for different purposes. The .json files of the communities crawled can be viewed in the supporting information. One researcher executed the crawling on a single PC for 48 ~ 72 hours, where the time varied with the size of the community. The Bitcoin and Ethereum forums were crawled on February 1 and 8, 2016, respectively, whereas the Ripple forum was crawled on January 21, 2016. Table 1 outlines the arrangement of the opinion data that were gathered.

**Table 1:** Summary of crawled opinion data.

Target Cryptocurrencies	Opinion Topics		
	Crawling Source	Crawling Boundary	Data Volume (threads)
Bitcoin	Bitcoin Forum	Dec. 01, 2013~ Feb. 01, 2016	13,360
Ethereum	Ethereum Forum	Aug. 07, 2015~ Feb. 08, 2016	1,449
Ripple	Ripple Forum	Sept. 07, 2015~ Jan. 21, 2016	468

The crawled data included garbage, e.g., ads and meaninglessly repetitive postings or replies. Quite a few spam filtering techniques were investigated to remove such garbage data [15, 24–29]. Any posting of more than two sentences found more than five times a day was considered spam and treated as such.

### Tagging user comments data

In this step, positive/negative replies to the crawled user comment data were tagged. Many past studies have dealt with classifying user sentiment or comment data [15, 30–35]. In this vein, user reviews have been used to create a classifier based on machine learning [36–40], and user comments on the Web have been statistically analyzed for sentiment tagging [41–43].

Past research has mostly focused on classifying user comments in particular fields. Comments on online communities involve considerable use of neologisms, slang, and emoticons that transcend grammatical usage. C.J. Hutto and Eric Gilbert introduced an algorithm called VADER [44] to parse such expressions, and proposed a method to analyze social media texts by drawing on a rule-based model. Online communities of interest in this paper paralleled social media texts. Thus, user comment data were tagged based on this algorithm.

VADER normalizes positive and negative sentiments from -1 to 1. Based on the normalized figure, x, -1< = x < -0.6, -0.6< = x < -0.2, 0.2 < = x <0.6, and 0.6< = x < = 1.0 were tagged as very negative, negative, positive, and very positive, respectively. In this paper, each of the comments and replies was tagged (see the opinion analysis example in Table 2).

**Table 2:** Bitcoin Community Opinion Analysis Example.

Opinion Criteria	Example topic sentences
Very Positive	“I am selling for $100 a Starbucks Gift card with a loaded balance of $20 worth of BTC” / “Bitcoin is the global currency of the Earth” / “How can 1 BTC eventually be worth $11 M”
Positive	“We are in Bitcoin Heaven” / “Bitcoin to eventually replace Apps like Uber” / “Russians can Pay Internet and phone bills with Bitcoin without fees”
Neutral	“Do you think Bitcoin will disappear or sopt being used?” / “What you like the best about Bitcoin?” / “Can Bitcoin make banks disappear?”
Negative	“Bitcoin: Should you stay or should you go?” / “Is there a way to earn at least $1 in BTC per hour?” / “IMF fears cryptocurrencies may circumvent capital controls”
Very Negative	“Bitcoin used to be involved in money laundering—will it become a huge problem?” / “Bitcoin cold storage—Hacked easily” / “Russia's Finance Ministry wants to ban Bitcoin”

### Prediction modeling

The crawled user comment data were tagged to create a prediction model. To create the prediction model, data selection was performed again. All opinions from very negative to very positive comments and replies could have been used. Yet, we intended to improve the qualitative results and minimize operation cost. For data selection, we performed an association analysis between the results of opinion analysis and fluctuations in cryptocurrency prices. In this paper, the Granger causality test, which is widely used in research on the value of shares and currencies, was adopted [45].

**Fig 2:**
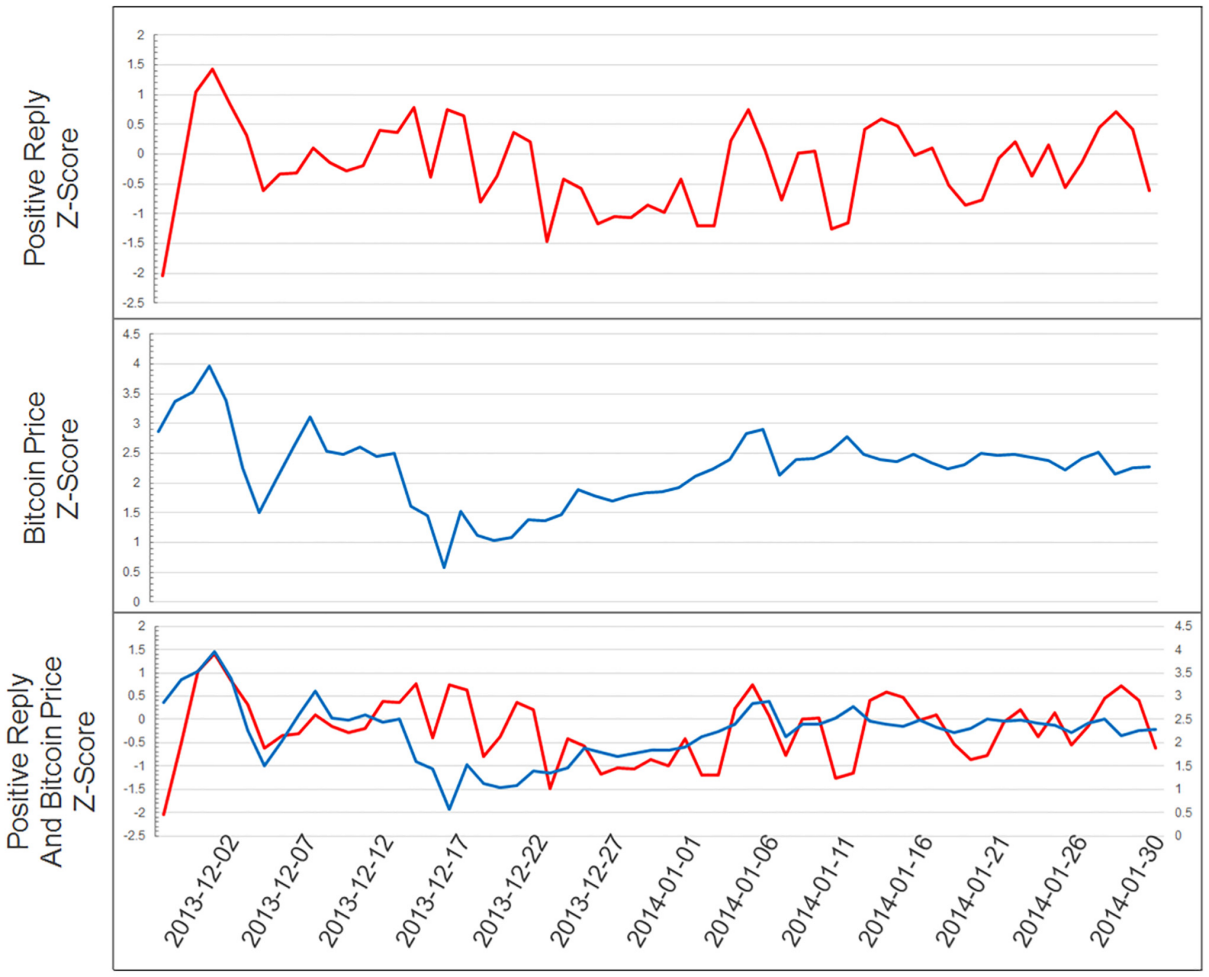
Z-scores of fluctuations in cryptocurrency prices overlapping with results of opinion analysis. Some opinions show a trend similar to that of fluctuations in cryptocurrency prices.

As shown in Eq 1, the results of opinion analysis based on the topics and replies (VADER-based tagged values), the number of topics posted, the number of replies posted, and the number of views of the entire topics posted on a certain day were transformed into z-scores for standardization against the previous 10 days. Likewise, the fluctuations in the price and number of transactions of cryptocurrencies were transformed into z-scores for standardization against the previous 10 days. On a certain date t (t = 10 in the paper), the z-score of a certain item E, denoted by $Z_{E}$, was defined as:

$$Z_{E_{t}}=\frac{E-\bar{x}\left(E\right)}{\sigma \left(E\right)}$$ (1)

where $\bar{x}\left(E\right)$ and σ(E) respectively represent the mean and standard deviation of each item for every date. Fig 2 shows an example of test results comparing the fluctuations in cryptocurrency prices and results of opinion analysis z-scores.

The standardized z-scores underwent the Granger causality test to determine the significance of association. The Granger causality test relies on the assumption that if a variable X causes Y, then changes in X will systematically occur before changes in Y [46]. As demonstrated in previous studies, lagged values of X exhibit a statistically significant correlation with Y [15, 46]. Correlation does not prove causation, however. We are not testing actual causation, but only whether the time series of a community of opinions contained predictive information regarding the fluctuations in cryptocurrency prices.

Our time series for the prices of cryptocurrencies and number of transactions, denoted by *S*_*t*_, reflected daily changes in the prices of cryptocurrencies and the number of transactions. To test whether the community opinions in the time series can predict changes in the fluctuations in cryptocurrency prices, we compared the variance explained by two linear models, as shown in Eqs 2 and 3. The first model uses only n lagged values of *S*_*t*_ (i.e., *S*_*t*−1_, ⋯, *S*_*t*−*n*_) for prediction, whereas the second model uses the n lagged values of both *S*_*t*_ and the selling prices of the item time series, denoted by *X*_*t*−1_, ⋯, *X*_*t*−*n*_. We performed the Granger causality test according to models in Eqs 2 and 3.

$$S_{t}=\alpha +\sum_{i=1}^{n}\beta_{i}S_{t-i}+\epsilon_{t}$$ (2)

$$S_{t}=\alpha +\sum_{i=1}^{n}\beta_{i}S_{t-i}+\sum_{i=1}^{n}\gamma_{i}X_{t-i}+\epsilon_{t}$$ (3)

Based on the results of the Granger causality test, we can reject the null hypothesis, whereby the community opinions time series does not predict fluctuations in cryptocurrency prices—i.e., *β*_{1,2,⋯,*b*}_ ≠ 0—with a high level of confidence The community opinions with the highest Granger causality relation (p-value < 0.05) were extracted.

The Granger causality test was performed on each currency for a time lag of 1 to 13 days. Experimentally, a time lag of 14 days and longer proved insignificant. Depending on the difference in each time lag measurement, elements showing significant associations were identified. For the prediction, the fluctuations in cryptocurrency prices were determined in a binary manner. We generated and validated the prediction model based on averaged one-dependence estimators (AODE) [47]. Based on AODE, we estimated the probability of a binary class *y*, given that an item-related set of features was *x*_1_,⋯*x*_*n*_, P(y|*x*_1,_⋯*x*_*n*_). This probability was estimated as follows:

$$\hat{P}\left(y|x_{1},\cdots x_{n}\right)=\frac{\sum_{i:1\leq i\leq n\wedge F\left(x_{i}\right)\geq m}\hat{P}\left(y,\;x_{i}\right)\prod_{j=1}^{n}\hat{P}(x_{j}|y,x_{i})}{\sum_{y\prime \in Y}\sum_{i:1\leq i\leq n\wedge F\left(x_{i}\right)\geq m}\hat{P}\left(y^{\prime },\;x_{i}\right)\prod_{j=1}^{n}\hat{P}(x_{j}|y^{\prime },x_{i})}$$ (4)

where $\hat{P}\left(\cdot \right)$ denotes an estimate of *P*(⋅), *F*(⋅) is the frequency, and *m* is the frequency limit set at 1 in this paper. In the next section, we discuss the results of the applied system.

## Experimental Results

Using our model, we made predictions regarding three cryptocurrencies (Bitcoin, Ethereum, and Ripple). In consonance with the days for which data were collected from these communities, each cryptocurrency’s daily price and number of transactions were crawled. Information concerning the price and number of transactions of Bitcoin was crawled via Coindesk [19], whereas price information for Ethereum was crawled via CoinMarketCap [22] and its transaction information was crawled via Etherscan [48]. Information regarding price for Ripple was crawled via rippleCharts [49], whereas its transaction information was not crawled. All data collected were in the public domain and excluded personal information. Table 3 outlines the arrangement of the market data that were gathered.

**Table 3:** Summary of crawled market data.

Target Cryptocurrencies	Cryptocurrency prices			Cryptocurrency transactions		
	Crawling Source	Crawling Boundary	Data Volume (days)	Crawling Source	Crawling Boundary	Data Volume (days)
Bitcoin	CoinDesk	Dec. 01, 2013~ Feb. 01, 2016	793	CoinDesk	Dec. 01, 2013~ Feb. 01, 2016	793
Ethereum	CoinMarketCap	Aug. 07, 2015~ Feb. 08, 2016	187	Etherscan	Aug. 07, 2015~ Feb. 08, 2016	187
Ripple	rippleCharts	Sept. 07, 2015~ Jan. 21, 2016	137			

The elements that exhibited significant associations in modeling for predictions were used for learning (Tables 4–8). P-values in the table are only shown for elements with prices of 0.05 or less.

**Table 4:** Statistical significance (p-values) of bivariate Granger causality correlation for Bitcoin price and community opinion.

Time Lag	Bitcoin Price										
	Very Positive	Positive	Neutral	Very Positive Reply	Positive Reply	Neutral Reply	Negative Reply	Very Negative Reply	Topic	Views	Reply
1 day	0.2318	**0.0007**	0.0753	0.2555	**0.0221**	0.3269	0.1237	0.126	**0.0406**	**0.0086**	0.1107
2 days	0.712	**0.0099**	0.0934	0.6289	**0.0354**	0.3436	0.312	0.1212	0.2158	0.4940	0.1709
3 days	0.7725	**0.0224**	**0.0173**	0.6916	0.075	0.5384	0.0811	0.1995	**0.0456**	0.0652	0.2459
4 days	**0.0044**	**0.0009**	**0.0158**	**0.0051**	**0.00004**	**0.0325**	**0.0021**	**0.0122**	**0.0009**	**0.0077**	**0.0006**
5 days	**0.00001**	**0.0021**	**0.0131**	**0.0034**	**0.00002**	**0.0081**	**0.0051**	**0.0196**	**0.00007**	**0.0029**	**0.0005**
6 days	**0.0004**	**0.0080**	0.0517	**0.0385**	**0.0008**	0.0771	**0.0431**	0.0884	**0.0046**	**0.0169**	**0.0118**
7 days	**0.0002**	**0.0117**	**0.0252**	0.0605	**0.0017**	0.1235	**0.0352**	**0.0286**	**0.0012**	**0.0229**	**0.0130**
8 days	**0.0005**	**0.0255**	**0.0306**	0.0901	**0.0039**	0.2943	0.0671	0.0508	**0.0050**	**0.0292**	**0.0247**
9 days	**0.0011**	**0.0065**	0.0512	0.0885	**0.0072**	0.1983	0.0678	0.0695	**0.0070**	**0.0328**	**0.0289**
10 days	**0.0026**	**0.0200**	0.0866	0.0793	**0.0051**	0.1862	**0.0331**	**0.0363**	**0.0092**	**0.0141**	**0.0189**
11 days	**0.0011**	**0.0337**	0.1265	0.0882	**0.0058**	0.1964	0.0628	**0.0317**	**0.0063**	**0.0316**	**0.0024**
12 days	**0.0024**	**0.0412**	0.1382	**0.0378**	**0.0030**	0.117	**0.0412**	**0.0261**	**0.0073**	0.0774	**0.0109**
13 days	**0.0019**	0.0615	0.1093	**0.0411**	**0.0058**	0.1783	**0.0328**	**0.0368**	**0.0061**	0.0893	**0.0149**

**Table 5:** Statistical significance (p-values) of bivariate Granger causality correlation for the number of transactions and community opinion for Bitcoin.

Time Lag	Bitcoin Transaction										
	Very Positive	Positive	Neutral	Negative	Very Negative	Very Positive Reply	Positive Reply	Neutral Reply	Topic	Views	Reply
1 day	**0.0003**	**0.0290**	**0.0025**	0.1524	**0.0177**	0.198	0.6988	0.0775	**0.0002**	**0.0036**	0.647
2 days	**0.000003**	**0.0374**	**0.0001**	**0.0146**	**0.0177**	0.2801	**0.0494**	**0.0124**	**0.000009**	**0.0011**	0.1362
3 days	**0.00007**	**0.0022**	**0.0008**	**0.0402**	0.0641	0.3693	0.1508	0.0558	**0.0001**	**0.0025**	0.2696
4 days	**0.0015**	**0.0099**	**0.0067**	**0.0247**	0.1808	0.6088	0.3392	0.217	**0.0017**	**0.0153**	0.5221
5 days	**0.0086**	**0.0363**	**0.0434**	0.0815	0.4	0.3906	0.2921	0.7686	**0.0048**	0.0869	0.4328
6 days	**0.0072**	0.1135	0.1654	**0.0364**	0.5244	**0.0050**	**0.0145**	0.0969	**0.0023**	0.3711	**0.0398**
7 days	**0.0073**	0.0733	0.3251	0.071	0.524	**0.0021**	**0.0283**	0.1575	**0.0072**	0.6176	0.0711
8 days	**0.0161**	0.2287	0.3298	**0.0284**	0.1864	**0.0099**	0.0613	0.3123	**0.0014**	0.4865	0.0965
9 days	**0.0245**	0.1897	0.0971	**0.0451**	0.2364	**0.0045**	**0.0412**	0.2797	**0.0019**	0.4004	0.0848
10 days	**0.0209**	0.1997	0.0882	**0.0253**	0.3111	**0.0053**	0.061	0.3635	**0.0020**	0.5301	0.111
11 days	**0.0288**	0.0764	0.1129	**0.0345**	0.393	**0.0043**	0.0602	0.3847	**0.0016**	0.6303	0.0883
12 days	**0.0457**	0.1615	0.1176	0.0531	0.4839	**0.0107**	0.0743	0.4382	**0.004**	0.735	0.1136
13 days	0.0763	0.224	0.1533	0.0694	0.5463	**0.0225**	0.0984	0.405	**0.0082**	0.82	0.1376

**Table 6:** Statistical significance (p-values) of bivariate Granger causality correlation for Ethereum’s price and community opinion.

Time Lag	Ethereum Price												
	Very Positive	Positive	Neutral	Negative	Very Negative	Very Positive Reply	Positive Reply	Neutral Reply	Negative Reply	Very Negative Reply	Topic	Views	Reply
1 day	**0.0106**	0.5194	0.8892	**0.0009**	0.9790	**0.0011**	**0.0232**	**0.0103**	0.2911	0.0840	0.0974	**0.0003**	**0.0085**
2 days	0.0799	0.9954	0.2773	**0.0325**	0.0558	0.1806	0.1727	0.2943	0.2195	0.2452	0.0769	0.6574	0.1837
3 days	0.2131	0.7819	0.1604	0.1658	0.1154	0.4765	0.0620	0.3496	**0.0179**	0.3592	0.0619	0.6498	0.0578
4 days	0.2928	0.5582	0.2006	0.0837	**0.0210**	0.3964	**0.0010**	0.3584	**0.0014**	0.4483	0.0934	0.3554	**0.0139**
5 days	0.3940	0.4873	0.2616	**0.0402**	**0.0012**	0.1372	**0.0002**	0.1994	**0.0031**	0.4136	0.2316	0.2981	**0.0051**
6 days	0.3688	0.3359	0.2039	0.0691	**0.0016**	0.0973	**0.0004**	0.2107	**0.0064**	0.1984	0.0809	0.1497	**0.0086**
7 days	0.3222	**0.0006**	0.0931	**0.0019**	**0.0011**	**0.0270**	**0.0002**	0.0885	**0.0002**	**0.0367**	0.0640	0.0680	**0.0043**
8 days	**0.0169**	**0.0054**	**0.0079**	**0.0011**	**0.0026**	**0.0343**	**0.0060**	0.0808	**0.0052**	**0.0115**	**0.0272**	0.0935	**0.0144**
9 days	0.2228	**0.0132**	0.0653	**0.0207**	**0.0166**	0.1008	**0.0041**	0.4138	**0.0332**	**0.0496**	0.1582	0.4450	0.1692
10 days	0.3766	0.0620	0.2518	**0.0148**	**0.0121**	0.1903	**0.0058**	0.2417	0.0692	0.2001	0.4131	0.7560	0.2621
11 days	0.5807	0.1346	0.3290	**0.0352**	**0.0177**	0.2414	**0.0118**	0.3994	0.1257	0.3621	0.5574	0.8875	0.3475
12 days	0.6158	0.1178	0.2648	**0.0458**	**0.0190**	0.1347	**0.0120**	0.3421	0.1488	0.2285	0.3906	0.4962	0.3025
13 days	0.2783	0.1923	0.2048	**0.0410**	**0.0157**	0.2731	**0.0070**	0.3773	0.0585	0.0778	0.6500	0.4462	0.3243

**Table 7:** Statistical significance (p-values) of bivariate Granger causality correlation for the number of transactions and community opinion for Ethereum.

Time Lag	Ethereum Transaction		
	Positive	Negative	Very Negative Reply
1 day	**0.0460**	**0.0248**	0.0567
11 days	0.6142	0.9875	**0.0179**
12 days	0.6358	0.9942	**0.0292**
13 days	0.6814	0.9959	**0.0385**

**Table 8:** Statistical significance (p-values) of bivariate Granger causality correlation for Ripple’s price and community opinion.

Time Lag	Ripple Price		
	Negative	Very Negative	Negative Reply
1 day	0.0781	**0.0033**	0.3903
2 days	0.1951	**0.0138**	0.2366
3 days	0.2649	**0.0150**	0.2033
4 days	0.3413	**0.0322**	0.0659
5 days	0.3228	**0.0124**	**0.0374**
6 days	0.3841	**0.0155**	0.0539
7 days	**0.0450**	**0.0185**	**0.0380**
8 days	0.0677	**0.0320**	**0.0339**
9 days	0.0826	0.0557	**0.0051**
10 days	0.0699	0.0880	**0.0064**
11 days	0.0985	0.0983	**0.0068**
12 days	**0.0272**	0.1464	**0.0106**
13 days	**0.0091**	0.1921	**0.0112**

An example of applicable input data is shown in Table 9. The results of the predicted fluctuations in the price and number of transactions of each cryptocurrency are discussed below.

**Table 9:** Example of a machine learning dataset. The z-score ($Z_{E_{t}}$) of data for the previous 10 days was used as the values A~J, which indicate the value of the sum of the opinion of each community at the given date. Here, X~Z indicate the topic data values (number of topics, sum of replies, sum of views) on the given date.

Data Class	Date	Opinion Data										Topic Data		
		Very Positive Topic	Positive Topic	Neutral Topic	Negative Topic	Very Negative Topic	Very Positive Reply	Positive Reply	Neutral Reply	Negative Reply	Very Negative Reply	Number of Topics	Sum of Replies	Sum of Views
Crawled Raw Data	Jan 02, 2016	A	B	C	D	E	F	G	H	I	J	X	Y	Z
Input Learning Data	Jan 02, 2016	$Z_{{\text{A}}_{t}}$	$Z_{{\text{B}}_{t}}$	$Z_{{\text{C}}_{t}}$	$Z_{D_{t}}$	$Z_{{\text{E}}_{t}}$	$Z_{{\text{F}}_{t}}$	$Z_{{\text{G}}_{t}}$	$Z_{{\text{H}}_{t}}$	$Z_{{\text{I}}_{t}}$	$Z_{{\text{J}}_{t}}$	$Z_{{\text{X}}_{t}}$	$Z_{{\text{Y}}_{t}}$	$Z_{{\text{Z}}_{t}}$

The accuracy rate, the F-measure and the Matthews correlation coefficient (MCC) were used to evaluate the performance of the proposed models. The computation of these evaluation measures required estimating precision and recall, which are evaluated from True Positive (TP), False Positive (FP), True Negative (TN) and False Negative (FN). These parameters are defined in Eqs 5, 6, 7 and 8:

$$Precision_{up}=\frac{TP}{TP+FP}$$ (5)

$$Precision_{down}=\frac{TN}{TN+FN}$$ (6)

$$Recall_{up}=\frac{TP}{TP+FN}$$ (7)

$$Recall_{down}=\frac{TN}{TN+FP}$$ (8)

Accuracy rate, weighted average of F-measure (*F*−*Measure*_*w*_) and MCC are defined in Eqs 9, 10, 11, 12 and 13.

$$Accuracy=\frac{TP+TN}{TP+FP+TN+FN}$$ (9)

$$F-measure_{up}=\frac{2\times Precision_{up}\times Recall_{up}}{Precision_{up}+Recall_{up}}$$ (10)

$$F-measure_{down}=\frac{2\times Precision_{down}\times Recall_{down}}{Precision_{down}+Recall_{down}}$$ (11)

$$F-measure_{w}=\frac{\left(F-measure_{up}\times \left(TP+FP\right)\right)+\left(F-measure_{down}\times \left(TN+FN\right)\right)}{TP+FP+TN+FN}$$ (12)

$$MCC=\frac{TP\times TN-FP\times FN}{\sqrt{\left(TP+FP\right)\left(TP+FN\right)\left(TN+FP\right)\left(TN+FN\right)}}$$ (13)

Of the Bitcoin-related data for 793 days, the first 88% (for 697 days) and the remaining 12% (for 94 days) were used for learning and verification, respectively. Fluctuations in the price of Bitcoin proved to be significantly associated with the number of topics, positive/very positive comments, and positive replies. The prediction result proved to be the highest when the time lag was six days with an accuracy of 79.57% (Table 10). Moreover, fluctuations in the number of transactions proved to be significantly associated with the section where a number of daily topics, very positive comments, and very positive replies were found. The predicted result of fluctuating numbers of transactions proved to be highest when the time lag was three days with an accuracy of 77.895% (Table 10).

**Table 10:** Experimental result of predicted Bitcoin fluctuation.

Time Lag	Bitcoin Price			Bitcoin Transaction		
	Accuracy(%)	F1-Score	MCC	Accuracy(%)	F1-Score	MCC
1 day	51.579	0.521	0.067	61.053	0.610	0.212
2 days	54.737	0.547	0.096	64.211	0.638	0.233
3 days	49.474	0.497	0.010	**77.895**	0.774	0.579
4 days	55.319	0.552	0.102	72.340	0.719	0.486
5 days	65.957	0.656	0.321	48.936	0.495	-0.048
6 days	**79.570**	0.796	0.606	42.553	0.426	-0.162
7 days	60.638	0.597	0.216	52.128	0.514	0.028
8 days	55.319	0.552	0.105	63.830	0.634	0.283
9 days	67.021	0.668	0.320	59.574	0.595	0.192
10 days	51.064	0.512	0.024	56.383	0.565	0.121
11 days	57.447	0.574	0.154	50.000	0.506	-0.021
12 days	49.462	0.495	-0.011	45.161	0.449	-0.121
13 days	50.538	0.506	0.012	48.387	0.489	-0.040

A 10-fold cross-validation was performed on Ethereum for the entire days (for 187 days). Unlike Bitcoin, Ethereum showed a significant association in the Granger causality test with the section where a number of negative/very negative comments were found. A significant association with a number of positive user replies was also found. The predicted result proved to be highest when the time lag was six days with an accuracy of 71.823% (Table 11). The fluctuation in the number of transactions showed insignificant associations with most sections, but was significantly associated with very negative replies when the time lag was 11~13 days. The predicted fluctuation in the number of transactions when the time lag was one day yielded an accuracy of 66.129% (Table 11).

**Table 11:** Experimental result of predicted Ethereum fluctuation.

Time Lag	Ethereum Price			Ethereum Transaction		
	Accuracy(%)	F1-Score	MCC	Accuracy(%)	F1-Score	MCC
1 day	53.763	0.533	0.058	**66.129**	0.661	0.315
2 days	52.432	0.524	0.042			
3 days	45.652	0.456	-0.095			
4 days	54.645	0.546	0.086			
5 days	51.381	0.514	0.021			
6 days	**71.823**	0.717	0.430			
7 days	63.333	0.633	0.259			
8 days	67.039	0.669	0.331			
9 days	49.438	0.490	-0.030			
10 days	49.718	0.496	-0.016			
11 days	55.682	0.555	0.103	64.205	0.641	0.276
12 days	50.286	0.501	-0.006	54.286	0.543	0.079
13 days	49.425	0.495	-0.013	51.149	0.512	0.020

Finally, Ripple underwent 10-fold cross-validation for the entire days (for 137 days). The predicted fluctuation in the price of Ripple proved to be highest when the time lag was seven days with an accuracy of 71.756% (Table 12).

**Table 12:** Experimental result of predicted Ripple price fluctuation.

Time Lag	Ripple Price		
	Accuracy(%)	F1-Score	MCC
1 day	61.314	0.613	0.206
2 days	50.735	0.510	0.013
3 days	51.852	0.517	0.011
4 days	52.593	0.528	0.055
5 days	62.406	0.624	0.236
6 days	42.424	0.426	-0.153
7 days	**71.756**	0.704	0.431
8 days	53.077	0.530	0.049
9 days	50.388	0.496	-0.025
10 days	60.938	0.610	0.210
11 days	63.780	0.638	0.268
12 days	53.157	0.527	0.040
13 days	63.200	0.628	0.243

Like Ethereum, Ripple proved to be significantly associated with very negative comments, and with negative replies when the time lag was seven days and longer. The prediction of fluctuation in the number of transactions of Ripple could not be performed due to difficulties in acquiring relevant data.

To determine the effectiveness of the proposed prediction model, we performed a simulated investment in Bitcoin, using the simulated investment technique generally used in past studies on stock price prediction [50]. We invested in Bitcoin when the model predicted the price would rise the following day, and did not invest when the price was expected to drop the following day according to the model. The simulated investment was based on the rule whereby we would gain or lose from the investment (m) by r, which indicates the increment or decrement in the Bitcoin price (m = m + m × r or m = m−m × r, respectively). The six-day time lag, which corresponded to the best result in this study, was used in the prediction model. The prediction model was created based on data for the period from December 1, 2013 to November 10, 2015. The 84-day or 12-week data for the period from November 11, 2015 to February 2, 2016 were used in the experiment.

**Fig 3:**
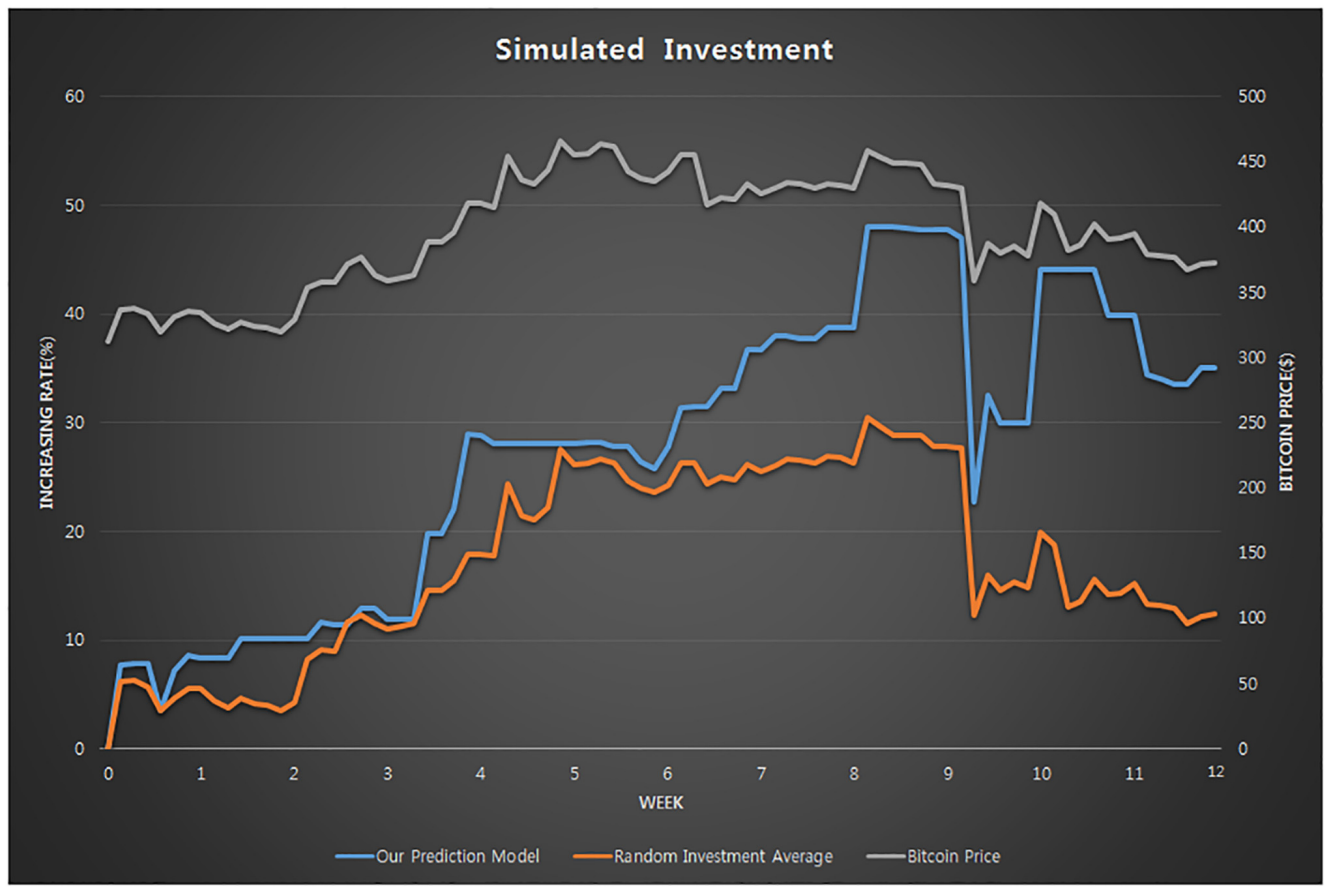
Increment/decrement in the amount of simulated investment in Bitcoin.

Fig 3 shows the results of the simulated investment program based on the above conditions. The random investment average refers to the mean of 10 simulated investments based on the random Bitcoin price prediction. Over 12 weeks, the Bitcoin price increased by 19.29% while the amount of investment grew by 35.09%. In random investment, the amount of investment increased by approximately 10.72%, which was lower than the increment in Bitcoin price.

## Discussion and Conclusion

This paper analyzed user comments in online communities to predict the price and the number of transactions of cryptocurrencies. The proposed method predicted fluctuations in the price of cryptocurrencies at low cost. In terms of the prediction rates for Bitcoin and other cryptocurrencies based on the limited resources in online communities, the proposed method paralleled previous studies designed for similar purposes [15, 51]. Moreover, user comments and replies in online communities proved to affect the number of transactions among users. The proposed method proved applicable to buying and selling cryptocurrencies, and shed light on aspects influencing user opinions. Furthermore, the simulated investment demonstrated that the proposed method is applicable to cryptocurrency trading.

Based on the learning data at the time of higher prediction rates, the types of comments that most significantly influenced fluctuations in the price and the number of transactions of each cryptocurrency were identified. Opinions affecting price fluctuations varied across cryptocurrencies. Positive user comments significantly affected price fluctuations of Bitcoin, whereas those of the other two currencies were significantly influenced by negative user comments and replies. Moreover, the association with the number of topics posted daily indicated that the variation in community activities could influence fluctuations in price. Further, unlike the price of cryptocurrencies, the number of transactions proved to be significantly associated with user replies rather than comments posted. Based on the prediction results, user opinions proved useful to predict the fluctuations in 6~7 days (Table 10).

The predicted fluctuations in the price of each cryptocurrency showed approximately 8% accuracy gaps. The predicted result was most precise in Bitcoin, which seems attributable to the amount of accumulated data and animated community activities (16.91 comments, 473.81 user replies, and 27443.18 views on average daily), which exerted a direct effect on fluctuations in the price of the cryptocurrency. The predicted result was least precise in Ripple, which had the smallest community regardless of its market size (3.41 comments, 29.14 user replies, and 1661.99 views on average daily). Ripple’s online community started in September, 2015, with little data accumulated and few user activities. These findings suggest that the difference in community sizes may have direct effects on fluctuations in the price of cryptocurrencies.

Improving the precision of prediction requires a few improvements. Despite the association analysis used to filter user comments and replies, more qualitative selection criteria are needed to build a prediction model. This paper focused on online communities to determine associations and predict fluctuations. Yet, as with past studies, using data on the Web [52, 53], analyzing social network data [46], and referring to search volumes on Google [10, 12] are conducive to more precise results. Moreover, partly adopting the stock market prediction technique used in previous studies [54] might help increase precision rate.

In this paper, we acquired information from users in online communities as a viable source for research on cryptocurrencies. In the same vein, the sentiments expressed by user comments and replies in online communities seem applicable to further analysis and understanding of cryptocurrencies. Moreover, the propensities of online community users may help understand the attributes of the relevant cryptocurrency. In addition, the rich information in online communities can contribute to understanding cryptocurrencies from different perspectives.

Cryptocurrencies are increasingly being used, and their usability has drawn attention from different perspectives [2–5]. Research on cryptocurrencies is insufficient, in that hardly any currency other than Bitcoin has been investigated. The proposed method of predicting fluctuations in the price and trading volume of cryptocurrencies based on user comments and replies in online communities is likely to increase the understanding and availability of cryptocurrencies if a range of improvements and applications are implemented. Furthermore, different approaches to user comments and replies in online communities are expected to bring more significant results in diverse fields.

## Supporting Information

S1 File Results of crawling Bitcoin forum, Ethereum forum, and Ripple forum (in .json format). (ZIP)

S2 File Python-based crawler source code for community data collection. (ZIP)

S1 Table The result of implementing opinion analysis from user opinion data (topic) on the Bitcoin forum (https://bitcointalk.org). (CSV)

S2 Table The result of implementing opinion analysis from user opinion data (topic) on the Ethereum forum (https://forum.ethereum.org/). (CSV)

S3 Table The result of implementing opinion analysis from user opinion data (topic) on the Ripple forum (http://www.xrpchat.com/). (CSV)

S4 Table The result of implementing opinion analysis from user opinion data (reply) on the Bitcoin forum (https://bitcointalk.org). (ZIP)

S5 Table The result of implementing opinion analysis from user opinion data (reply) on the Ethereum forum (https://forum.ethereum.org/). (CSV)

S6 Table The result of implementing opinion analysis from user opinion data (reply) on the Ripple forum (http://www.xrpchat.com/). (CSV)
